# Suitability of enterococcus faecalis as a test organism to evaluate in vivo efficacy of alcohol-based handrubs

**DOI:** 10.1186/2047-2994-4-S1-P307

**Published:** 2015-06-16

**Authors:** RA Leslie, DR Macinga, FH Brill, M Suchomel

**Affiliations:** 1 Research and Development, GOJO Industries, USA; 2 Northeastern Ohio Medical University, USA; 3 Dr. Brill + Partner GmbH- Institute for Hygiene and Microbiology, Germany; 4 Medical University Vienna, Austria

## Introduction

The World Health Organization has called for development of improved methods to evaluate efficacy of hand hygiene products that more closely reflect practical use patterns and have success criteria tied to clinical benefit. The test organisms should represent pathogens with environmental stability known for their potential to be spread by contaminated hands.

## Objectives

Determine the suitability of a non-pathogenic *Enterococcus faecalis* strain as an alternative to *Escherichia coli* for *in vivo* efficacy evaluation of alcohol-based handrubs (ABHR).

## Methods

Overall methodology based on European Norm 1500:2013. Test organisms were *E. faecalis*(ATCC 47077) or *E. coli* K12 (NCTC 10538). Hand contamination was performed by either the immersion method from EN 1500 or spreading 0.5 mL of a high titer suspension over all surfaces of both hands (based on ASTM E2755). Subjects performed a reference procedure according to EN 1500. Testing was conducted at two laboratories using *E. faecalis* and one facility using *E. coli*.

## Results

Mean log prevalues (PV) for *E. faecalis* contamination by immersion were 6.56±0.27 and 6.29±0.60 and mean log reduction factors (RF) were 5.68±0.92 and 4.63±0.26, at the respective labs. Mean log PV for *E. coli* contamination by immersion was 5.38±0.55 and mean log RF was 4.61±0.74. Mean log PVs for *E. faecalis* contamination by high titer were 5.78±0.40 and 6.82±0.09 and mean log RFs were 5.03±0.91 and 5.16±0.56, at the respective labs. Mean log PV for *E. coli* contamination by high titer was 4.76±0.52 and mean log RF was 3.86±0.95. *E. faecalis* yielded PVs that exceeded *E. coli* PVs by ~1-2 log units using either mode of contamination suggesting greater survival during hand drying.

## Conclusion

*E. faecalis* demonstrates strong potential as a test organism that represents a pathogen with high environmental stability known to be spread by contaminated hands in healthcare settings. Additionally, the low-volume contamination procedure is simpler to execute and more closely mimics the condition of hands when ABHR use is indicated in clinical settings (e.g. minimally soiled and fully dry). Further studies are needed to finalize the method and better understand the sensitivity of *E. faecalis* toalcohols to ensure it differentiates product performance similar to *E. coli*.

## Disclosure of interest

None declared.

